# Assessment of fungal spores and spore-like diversity in environmental samples by targeted lysis

**DOI:** 10.1186/s12866-023-02809-w

**Published:** 2023-03-14

**Authors:** Andrea Corona Ramirez, Danaé Bregnard, Thomas Junier, Guillaume Cailleau, Cristina Dorador, Saskia Bindschedler, Pilar Junier

**Affiliations:** 1 Laboratory of Microbiology, Institute of Biology, University of Neuchâtel, Neuchâtel, Switzerland; 2 Vital-IT Group, Swiss Institute of Bioinformatics, Lausanne, Switzerland; 3 Department of Biotechnology, University of Antofagasta, Antofagasta, Chile

**Keywords:** Fungi, Lysis-resistance, Extreme environment, Spores, Lake sediments, Microbial mats

## Abstract

At particular stages during their life cycles, fungi use multiple strategies to form specialized structures to survive unfavorable environmental conditions. These strategies encompass sporulation, as well as cell-wall melanization, multicellular tissue formation or even dimorphism. The resulting structures are not only used to disperse to other environments, but also to survive long periods of time awaiting favorable growth conditions. As a result, these specialized fungal structures are part of the microbial seed bank, which is known to influence the microbial community composition and contribute to the maintenance of diversity. Despite the importance of the microbial seed bank in the environment, methods to study the diversity of fungal structures with improved resistance only target spores dispersing in the air, omitting the high diversity of these structures in terms of morphology and environmental distribution. In this study, we applied a separation method based on cell lysis to enrich lysis-resistant fungal structures (for instance, spores, sclerotia, melanized yeast) to obtain a proxy of the composition of the fungal seed bank. This approach was first evaluated *in-vitro* in selected species. The results obtained showed that DNA from fungal spores and from yeast was only obtained after the application of the enrichment method, while mycelium was always lysed. After validation, we compared the diversity of the total and lysis-resistant fractions in the polyextreme environment of the Salar de Huasco, a high-altitude athalassohaline wetland in the Chilean Altiplano. Environmental samples were collected from the salt flat and from microbial mats in small surrounding ponds. Both the lake sediments and microbial mats were dominated by Ascomycota and Basidiomycota, however, the diversity and composition of each environment differed at lower taxonomic ranks. Members of the phylum Chytridiomycota were enriched in the lysis-resistant fraction, while members of the phylum Rozellomycota were never detected in this fraction. Moreover, we show that the community composition of the lysis-resistant fraction reflects the diversity of life cycles and survival strategies developed by fungi in the environment. To the best of our knowledge this is the first time that the fungal diversity is explored in the Salar de Huasco. In addition, the method presented here provides a simple and culture independent approach to assess the diversity of fungal lysis-resistant cells in the environment.

**Supplementary Information:**

The online version contains supplementary material available at 10.1186/s12866-023-02809-w.

## Introduction

The vast majority of fungal species form at a particular stage of their life cycle, single or multicellular specialized structures to reproduce or as a strategy to survive unfavorable environmental conditions. These structures are central in the life cycle of most fungal species. The most common strategy for survival or dispersal is the formation of spores. Fungal spores are specialized dormant cells that serve different purposes: reproduction, dispersal, and survival [1–3]. Accordingly, there is a large diversity of fungal spores that vary in shape, mode of formation, and state of dormancy, as well as in their level of stress resistance [4]. Many fungal species form different types of spores within their life cycle, with sexual spores being used for resistance and asexual spores for clonal dispersion. However, depending on the species, both types are known to be resistant to abiotic and biotic stress factors [5]. For example, the asexual spores (microconidia) of *Fusarium oxysporum* are as resistant to antifungals as vegetative cells [6], whereas the sexual spores of other species, like those of *Aspergillus spinosus*, *Paecilomyces variotii*, and *Talaromyces macrosporus,* show a heat-resistance similar to that of bacterial endospores [7].

Fungi are also remarkable in the morphological diversity of their vegetative phases, which may be unicellular, such as yeasts, or pluricellular, such as in the case of mycelial fungi. Some fungi can even transit from the yeast to the (pseudo)-hyphal stage, an ability termed dimorphism. This dimorphism is also part of their resistance to abiotic or biotic stresses. In this case, the yeast stage may be considered as an intermediate between spores and an actively metabolizing filamentous vegetative phase in regard to resistance. This is in particular the case of the so-called black fungi, which are considered poly-extremophiles and among the most stress-resistant eukaryotes on Earth [8, 9]. Finally, in addition to spores, fungi also form multicellular resistant structures called sclerotia that can remain latent in the environment for years. While some sclerotia are large and visible to the naked eye, other are minute structures that cannot be seen macroscopically (e.g., microsclerotia of *Verticillium* spp.) [10].

Up to now, the majority of studies about the taxonomic diversity of single or multicellular specialized fungal structures providing resistance in the environment have been focused on fungal spores suspended in the air [11–15]. On the other hand, studies on the fungal diversity in soils often target the diversity of specific functional groups (mycorrhiza or fungal pathogens) or the overall fungal diversity [16–21], but do not make a distinction between mycelium and other structures that enhance survival (such as spores or sclerotia). It is noteworthy to mention that a few studies have specifically targeted spore diversity of arbuscular mycorrhizal fungi (AMF) in soils by applying methods such as spore washing [22, 23]. This is feasible by the fact that AMF spores are large (40–800 µm) [24, 25] and thus easy to isolate by sieving. However, these methods are time consuming and susceptible to contamination during the multiple manipulation steps. As a result, generalized experimental procedures to specifically assess structures that promote survival in fungi in complex environments such as soil and sediments are currently inexistent.

Unicellular and multicellular specialized structures that promote survival can remain viable for long periods of time in the environment, becoming part of microbial seed banks, which have been shown to influence the microbial composition of an environment and to contribute to the maintenance of diversity [3, 26]. Thus, the development of more efficient methods that allow the assessment of this fraction in non-aerial samples would enable the study of fungal population shifts and the diversity of the fungal seed banks in soil, water, microbial mats, and other types of environmental samples. In this study, we assessed whether a method previously validated for the physical separation of bacterial endospores [27] could also be used to investigate the diversity of fungal structures involved in enhanced survival (spores, sclerotia, zoospores or zygospores) in the environment. In the case of bacteria, an important property of many known survival structures is their resistance to lysis. This was the basis to develop a method that uses physical and chemical treatments to lyse non-resistant cells and to enrich lysis-resistant structures [27]. When applied to bacterial communities, the procedure results in the enrichment of endospores from Firmicutes, but also lysis-resistant spores from other bacteria such as Actinobacteria or Cyanobacteria [28]. Even though in the case of the various structures promoting survival in fungi, their enhanced resistance to lysis has not been assessed systematically, previous studies have shown that lysis of survival structures such as spores requires harsher conditions [29], and that modifications in the spore wall composition and structure promote resistance [30]. Similarly, the modifications undergone during sclerotia tissue differentiation provide resistance to lysis [31]. Therefore, one could expect that the application of the procedure to enrich lysis-resistant cells would also result in the enrichment of fungal taxa able to form cellular structures with enhanced survival properties (herein referred to as spores and spore-like cells).

The use of the method for enriching lysis-resistant cells has been recently extended to study lysis-resistant communities in archaea. While in bacteria, lysis-resistance was shown to be restricted to a few groups, in archaea, resistance to lysis was widely spread [32]. This is likely due to specific cellular adaptations (i.e., lipid composition, diversity of cell envelope composition) within this domain [33–36]. Considering the diversity in morphology, function, formation, and resistance of fungal spores and spore-like cells, we hypothesized that, as in the case of Archaea, lysis-resistance will be widespread in fungi. This would reflect the multitude of morphological adaptations promoting survival in fungi. To test this hypothesis, we first validated the enrichment method for its application in fungi using mycelium-only biomass and resistant structures (sexual and asexual spores and yeast stages) from various clades: *Aspergillus niger, Ulocladium alternata*, *Coprinopsis cinerea, Mortierella antarctica,* and *Candida albicans.* These fungi were selected to represent different life cycles and types of specialized resistant structures. After validation, we applied the method to environmental samples, with the aim of assessing the abundance and diversity of fungal lysis-resistant structures. The samples were collected in the polyextreme environment of the Salar de Huasco. The Salar de Huasco is a high-altitude athalassohaline wetland located in the Chilean Altiplano at an altitude of 3,800 m.a.s.l. In addition to the high altitude, daily temperature changes from—10° to + 25 °C, high solar radiation < 1100 W/m^2^, and a negative water balance, are other extreme environmental conditions in this ecosystem [37, 38]. Furthermore, a high biodiversity of endemic bacteria and archaea has been reported for this salt flat [39–41], while the fungal diversity has never been explored. This is major knowledge gap in this ecosystem given that fungi are now recognized as key microbial players in extreme environments [42].

## Results

### Effect of the lysis-resistant enrichment method on pure cultures

Representatives of different fungal species were selected to produce a diversity of morphological structures including: non-melanized mycelium and asexual melanized spores of *A. niger* (Ascomycota); melanized mycelium and asexual spores of *U. alternata* (Ascomycota); non-melanized mycelium and sexual and asexual spores of *C. cinerea* (Basidiomycota); non-melanized mycelium and asexual spores of *M. antarctica* (Mucoromycota); and yeast cells of *C. albicans* (Ascomycota).

**Fig. 1:**
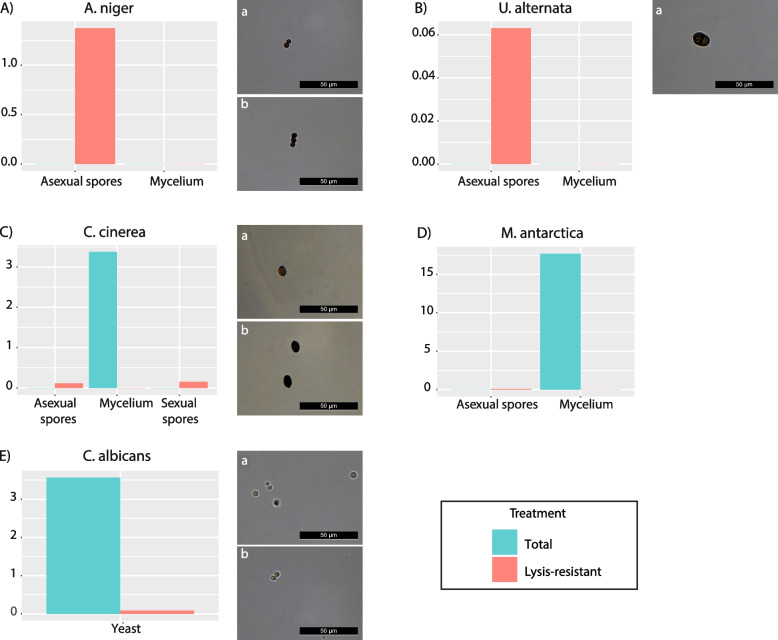
Validation of the lysis-resistant enrichment method. Bar plots showing the DNA concentration in ng/µl from the direct DNA extraction (“Total” in blue) and after the application of the method to enrich lysis-resistant cells (“Lysis-resistant” in red) for mycelium, sexual spores, asexual spores and/or yeast stage of different fungal species. **A** *Aspergillus niger*; **B** *Ulocladium alternata*; **C** *Coprinopsis cinerea*; **D** *Mortierella antarctica*; **E** *Candida albicans*. For **A**, **B**, **C**, and **E**: Microscopic images on the right show the spores before (**a**) or after (b) the lysis-enrichment method prior to DNA extraction. Due to the small number of spores after the enrichment method in *U. alternata* and the low concentration in *M.* *antarctica*, no microscopic images could be obtained from them

The resistance to lysis for each type of sample was evaluated by comparing the amount of DNA obtained after the method to enrich lysis-resistant cells to the DNA extracted directly (Additional Table 1). Our results show that the amount of DNA extracted depended on the type of sample (mycelium, spores or yeast stage), as well as on the type of extraction performed (DNA extraction after the enrichment treatment or without the enrichment). We arbitrarily chose to compare DNA yields from the extraction without normalization to initial biomass, as DNA concentrations per cell vary in Fungi [43, 44]. For *A*. *niger* and *U*. *alternata*, after the lysis-resistant enrichment treatment, DNA was obtained from spores only (Fig. 1A and B). In contrast no DNA was recovered from the mycelium samples with either extraction method. Similarly, a low amount (0.077 ng/μl and 0.111 ng/μl respectively) of DNA from the sexual and asexual spores of *C*. *cinerea*, was only recovered after the enrichment treatment, while mycelial DNA was only recovered with the direct DNA extraction method (Fig. 1C). Concerning *M*. *antarctica*, no DNA was recovered from the spores after the direct DNA extraction method, and only a very low amount of DNA (0.06 ng/μl) was recovered after the lysis-resistant enrichment method. Moreover, DNA from the mycelium samples was only obtained after the direct DNA extraction method (Fig. 1D). On the other hand, for *C*. *albicans*, which was only used under its yeast stage, a substantial amount of DNA (3.56 ng/μl) was recovered using the direct DNA extraction method, while only a small amount of DNA (0.086 ng/μl) was recovered using the lysis-resistance method (Fig. 1E). In agreement with the results obtained for the DNA quantification, microscopical images showed that spores of all species assessed can resist the enrichment method (Fig. 1). However, in the case of *M*. *antarctica*, the quantity of spores was too small to allow the generation of good optical microscopy images after the treatment.

### Fungal diversity in the Salar de Huasco

**Fig. 2:**
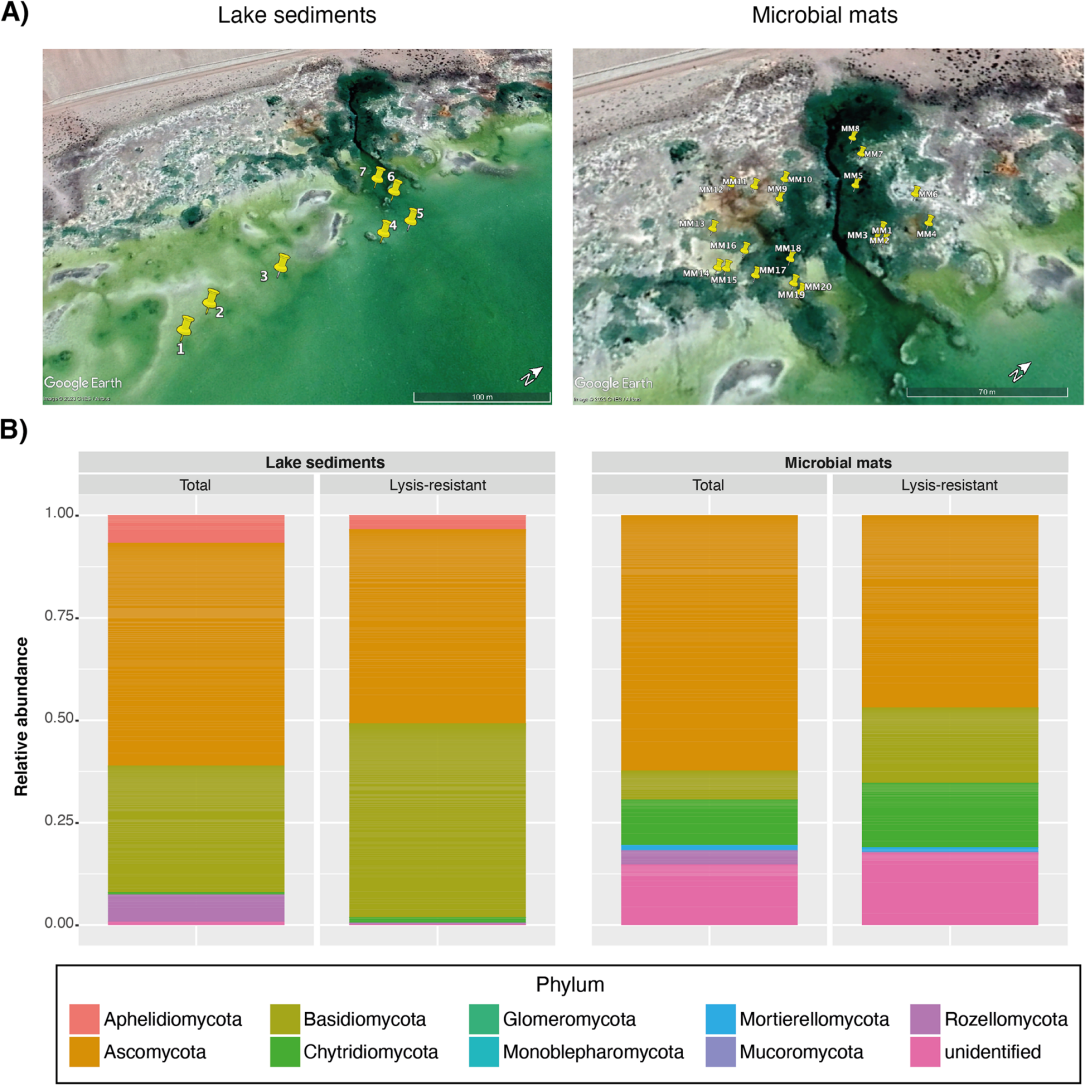
Fungal diversity at the Salar the Huasco. **A** Sampling zones indicating the position of the seven sediment cores collected from the main saline lake (right) and the ponds in which 20 microbial mat samples were collected (left). **B** Assessment of the fungal community composition per environment. Bar plots showing the fungal community composition at the phylum level, in the total community (DNA extraction from samples without enrichment) and lysis-resistant fraction (after lysis-resistant enrichment treatment) in the lake sediments (right) and microbial mats (left)

Samples were collected from two different environments at the Salar de Huasco: lake sediments from the main saline lake and microbial mats from small ponds surrounding the main lake (Fig. 2A). From the main saline lake, seven sediment core samples were collected along a salinity gradient ranging from 53 to 0.706 PSU. The pH in the lake ranged between 8.22 and 8.43 (not following a specific gradient). From the small ponds surrounding the main saline lake, 20 microbial mat samples were collected from 18 ponds randomly selected. The microbial mats varied in color, texture, and location. The pH in these ponds ranged between 7.67 and 9.74, and the salinity from 0.41 to 4.22 PSU. In both types of environments (lake sediments and microbial mats), the fungal diversity was assessed for the total fraction (i.e., DNA extraction without prior treatment to enrich lysis-resistant cells) and for the lysis-resistant fraction (i.e., after the application of the enrichment method).

Representatives of the Ascomycota and Basidiomycota were the most abundant in the total community fraction in both environments. Furthermore, representatives of Chytridiomycota were present at a higher relative abundance in the microbial mats than in the lake sediments, while the contrary was observed for Amplicon Sequence Variants (ASVs) assigned to the Rozellomycota phylum. ASVs assigned to the phyla Mucoromycota, Monoblepharomycota, and Glomeromycota, were only present in the microbial mats, but their relative abundance was low (below 0.04; Fig. 2B). Moreover, although representatives of the Aphelidiomycota phylum were present in both environments, their relative abundance in the microbial mats was below 0.0014 and is not visible in Fig. 2B. Another difference between the fungal communities in lake sediments and microbial mats was the large fraction of unidentified ASVs present in the latter, as compared to the lake sediments, in which the unidentified fraction, at the phylum level, is negligible. When comparing the overall composition of the fungal communities in the total and the lysis-resistant fractions, only a few differences could be observed. More specifically, the relative abundance of Basidiomycota and Chytridiomycota was higher in the lysis-resistant fraction, while the phylum Rozellomycota was only present in the total community of both types of environments.

**Fig. 3:**
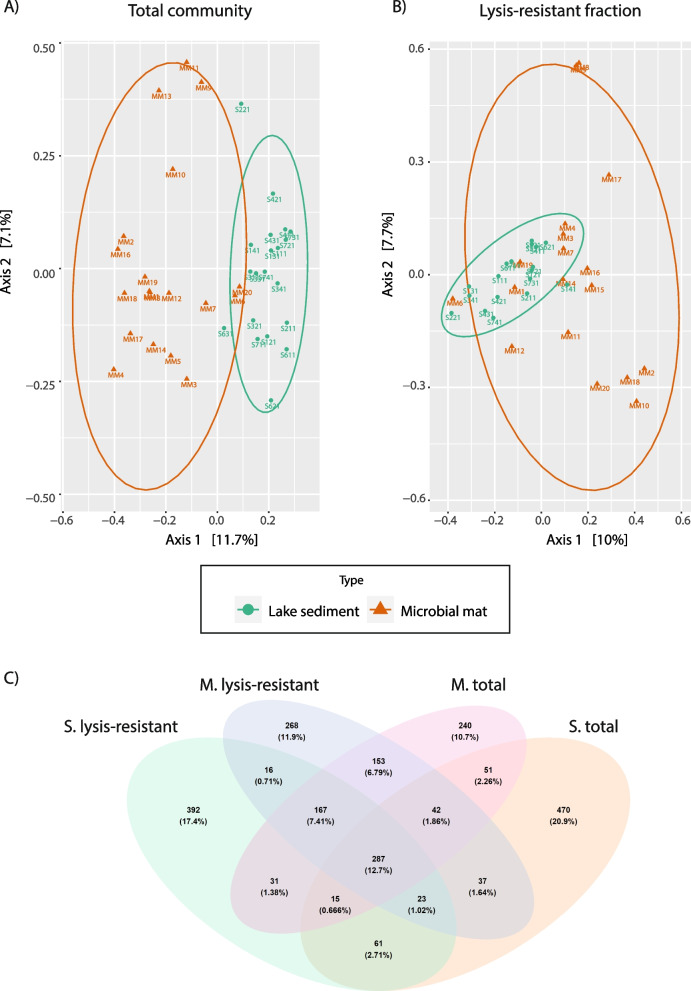
Principal Coordinates Analysis (PCoA) of the total community and the lysis-resistant fraction and Venn Diagram of the fungal community in the lake sediments and microbial mats. **A** PCoA calculated based on the Bray-Cutis distances of the total community, grouped by environment, lake sediments (green) and microbial mats (brown). **B** PCoA calculated based on the Bray-Cutis distances of the lysis-resistant fraction, grouped by environment, lake sediments (green) and microbial mats (brown). **C** Venn diagram of the top 1000 ASVs for each fraction and environment, microbial mat (M. total and M. lysis-resistant) and lake sediments (S. total and S. lysis-resistant)

From this first assessment, we decided to evaluate each environment separately, to reduce the effect of the environment type and to better assess the effect of the enrichment method on the characterization of the lysis resistant community. This decision was supported by a Principal Coordinates Analysis (PCoA) of the total community, which showed a clear separation of both environments (Fig. 3A), with the samples from microbial mats presenting a broader dispersion than those from the lake sediments. Moreover, the Bray–Curtis beta diversity between the two environments was statistically different (*p*-value 0.0202). In contrast, the PCoA analysis of the lysis-resistant community showed an overlap of the two environments (Fig. 3B) and the beta diversity was not statistically different (*p*-value 0.327). This indicated that the lysis-resistant fraction is partially similar in both environments. Accordingly, the Venn Diagram in Fig. 3C shows that 21.84% of the ASVs were shared among the lysis-resistant fraction (green and blue ovals) of both environments, whereas for the total community, only 17.49% of the ASVs were shared between the two environments (red and orange ovals).

### Diversity of lysis-resistant fungi in the Salar de Huasco

**Fig. 4:**
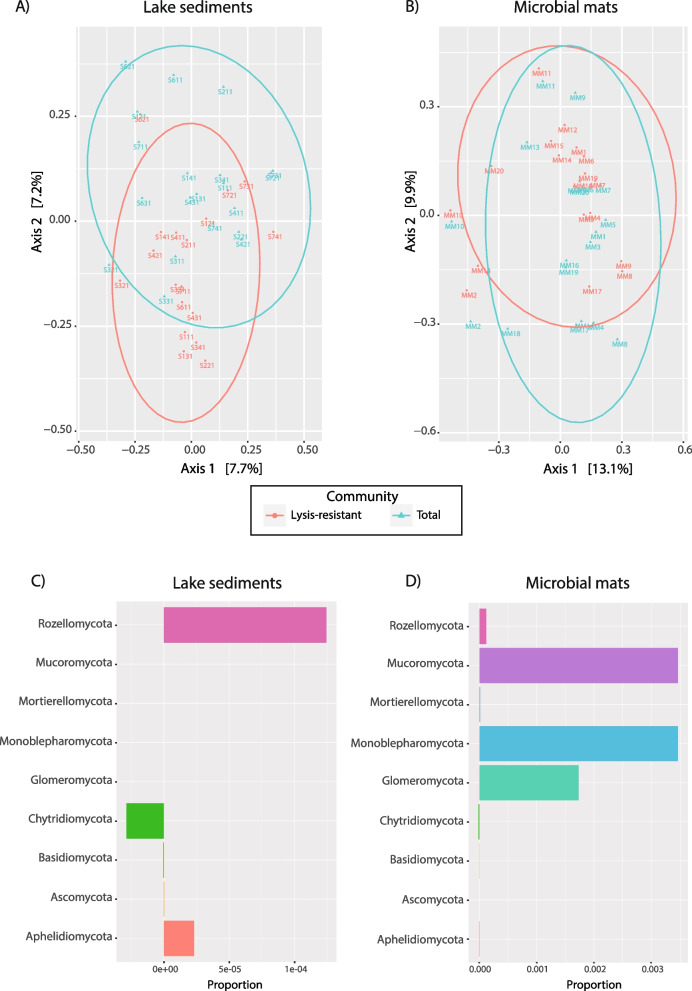
Principal Coordinates Analysis (PCoA) and bar plots representing the enrichment of different fungal phyla in the total versus lysis-resistant fractions per environment. PCoA calculated based on the Bray–Curtis distances of the communities in **A** lake sediments and **B** microbial mats for the lysis-resistant fraction (red) and the total community (blue). Enrichment per phyla in **C** lake sediments and **D** microbial mats in the lysis-resistant or total fraction. Negative values show enrichment in the lysis-resistant fraction, positive values show enrichment in the total fraction. Values close to zero show equal abundance in both fractions

The community analysis (PCoA) was performed using all samples, but for the comparison of the total *versus* the lysis-resistant fractions, only samples that could be paired (i.e., present in each fraction) were retained. For the lake sediments, sample 5 could not be analyzed as there was not enough sediment to perform the analysis. Furthermore, amplicons could not be generated from two samples of the lake sediments (total community 3.1.1 and 6.3.1) and from three samples of microbial mats (lysis-resistant fraction 5 and 13, and total community 15). These samples were removed to only consider paired samples (lysis-resistant *vs* total fraction) for the comparative analysis. A PCoA of the lake sediment and microbial mats is shown respectively in Fig. 4A and B. In both environments, the total community and lysis-resistant fractions overlapped. Not only was there no clear separation between the total community and the lysis-resistant fraction in the PCoA, but Shannon–Weaver and Simpson diversity indices calculated for the total community *versus* the lysis-resistant fraction were not statistically different (statistical difference in lake sediments p-value = 0.844 for the Shannon–Weaver index and *p*-value = 0. 0.939 for the Simpson index; in microbial mat *p*-value = 0.573 for the Shannon–Weaver index and *p*-value = 0.992 for the Simpson index).

#### Diversity at the phylum level

To better understand the effect of the enrichment method on the community composition, the relative enrichment of each phylum in the total *versus* the lysis-resistant fractions was plotted for the lake sediments (Fig. 4C) and the microbial mats (Fig. 4D). This analysis shows whether the ASVs belonging to a given phylum were more abundant in the total community (positive values), in the lysis-resistant fraction (negative values), or equally abundant (zero). Only five phyla were represent in the lake sediments, while nine phyla were represent in the microbial mats. In the lake sediments, only Chytridiomycota was enriched in the lysis-resistant fraction (Fig. 4C). The Basidiomycota phylum was slightly enriched in the lysis-resistant fraction, while the Ascomycota, Aphelidiomycota, and Rozellomycota were enriched in the total community. The Mucoromycota, Mortierellomycota, Monoblepharomycota and Glomeromycota phyla were only present in the microbial mats and were enriched in the lysis-resistant fraction (Fig. 4D). The phylum Chytridiomycota was slightly enriched in the lysis-resistant fraction in the microbial mats too. The Basidiomycota, Ascomycota, and Aphelidiomycota phyla show no enrichment in either fraction. These results reflect the differences observed in Fig. 2B when comparing the community composition between the two fractions and environments.

We then assessed the 50 most abundant ASVs at the phylum level. In the lake sediments, only the Chytridiomycota phylum was not among the 50 most abundant ASVs (Additional Fig. 1). The number of phyla observed in the microbial mats was higher. The 50 most abundant ASVs in the microbial mats belonged to the Ascomycota, Basidiomycota, Chytridiomycota, Mortierellomycota, and Rozellomycota phyla. Additionally, a large fraction belonged to unidentified ASVs (Additional Fig. 2). In both environments the phylum Rozellomycota was only present in the total community, while all other phyla were present in both fractions.

#### Diversity at the class level

**Fig. 5:**
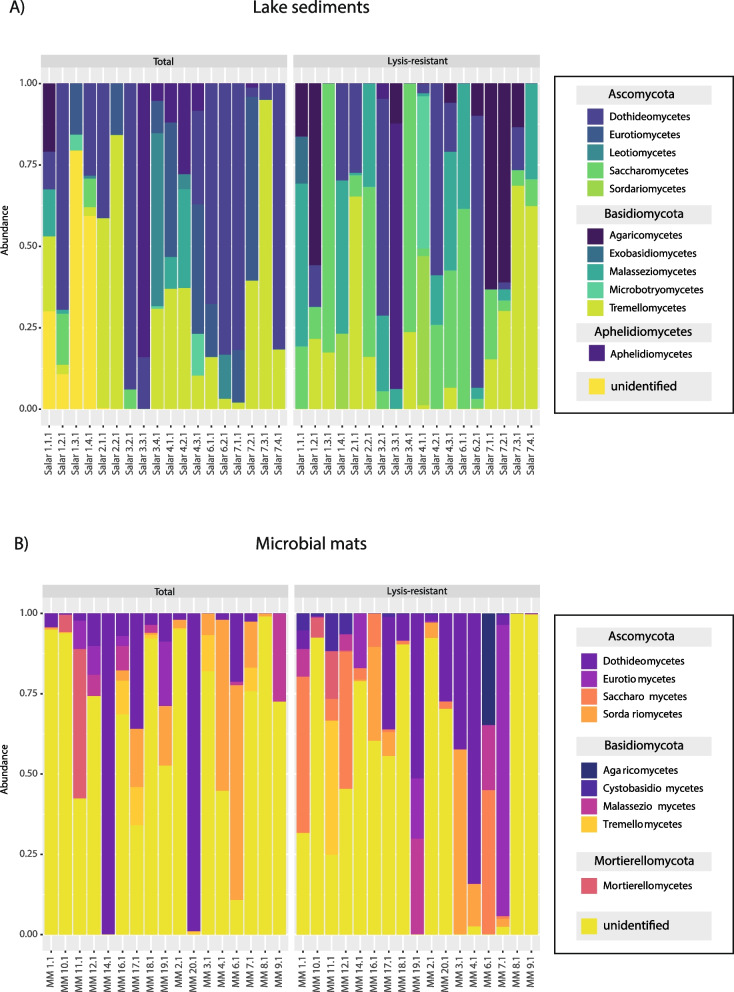
Bar plots showing the relative abundance of the 50 most abundant ASVs per sampling point in the **A** lake sediments and **B** microbial mats. Left the total community and right the lysis-resistant fraction. Different colors represent different classes, in the legend on the right the classes are classified by phyla

To better understand the differences in lysis-resistance of the fungal community, we assessed the same 50 most abundant ASVs at the class level in lake sediments (Fig. 5A) and microbial mats (Fig. 5B). These analyses show that the differences in lysis-resistance are found at lower taxonomic ranks. Additionally, these results also show the broad fungal diversity present in the Salar de Huasco. In the lake sediments, most of the classes were present in both fractions, except for the class Sordariomycetes (Ascomycota) and Exobasidiomycetes (Basidiomycota) that were only present in the lysis-resistant fraction, while the Eurotiomycetes (Ascomycota) and Leotiomycetes (Ascomycota) were only present in the total fraction (Fig. 5A). In the microbial mats, the classes Agaricomycetes (Basidiomycota), Cystobasidiomycetes (Basidiomycota), and Saccharomycetes (Ascomycota) were only found in the lysis-resistant fraction, while all other classes were present in both fractions (Fig. 5B). Furthermore, some differences were observed between the environments. For example, the classes Eurotiomycetes and Sordariomycetes that were only present in the lysis-resistant fraction in the lake sediments, were present in both fractions in the microbial mats. In contrast, the class Saccharomycetes was only present in the lysis-resistant fraction in the microbial mats, but was present in both fractions in the lake sediments. These results show the differential effect of the lysis-resistant enrichment method in fungi when comparing two different environments.

##### Diversity at the class level in the lake sediments

**Fig. 6:**
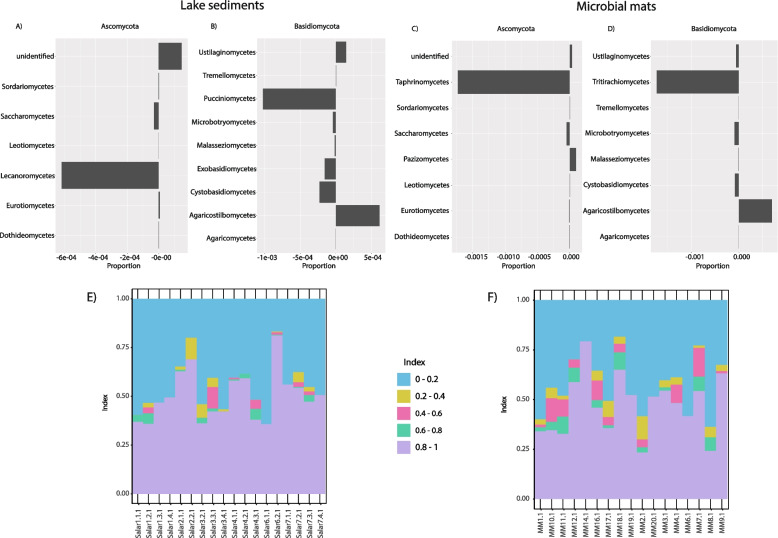
Enrichment of different fungal classes in the lysis-resistant or total fraction in the lake sediments and microbial mats and evaluation of the composition of the lysis-resistant community. Enrichment of the classes in the Ascomycota (**A**) and Basidiomycota (**B**) phyla in the lake sediments. Enrichment of the classes in the Ascomycota (**C**) and Basidiomycota (**D**) phyla in the microbial mats. Negative values show enrichment in the lysis-resistant fraction, positive values show enrichment in the total fraction, 0 indicates equal abundance in both fractions. The bar plots in the bottom of the Fig. 6 (**E**–**F**), show the fraction of the ASVs enriched in the lysis-resistant (enrichment index 0.8–1.0), the total community (enrichment index 0.0–0.2), or those shared between the two fractions in the lake sediments (**E**) and microbial mats (**F**). The enrichment index was calculated by comparing the abundance (sequence counts) in the lysis-resistant fraction over the total abundance (sequence counts in lysis-resistant fraction + total community)

Assessing the most abundant ASVs of a community leaves a large fraction of the diversity out of the analysis. Thus, the enrichment of all the different phyla at the class level was assessed separately. In the lake sediments, the only identified class within the Chytridiomycota phylum was Lobulomycetes. This class and unidentified sequences were enriched in the lysis-resistant fraction. Similarly, in the Rozellomycota phylum, the only class identified was the Rozellomycotina _cls_Incertae_sedis, which was enriched in the lysis-resistant fraction, while unidentified sequences were enriched in the total fraction. In the Aphelidiomycota phylum, the Aphelidiomycetes class (the only class described so far for this group) was enriched in the total fraction. Furthermore, in the Ascomycota phylum (Fig. 6A), only the Saccharomycetes and the Lecanoromycetes classes were enriched in the lysis resistant fraction. In the latter, two genera were present, the *Caloplaca* and *Acarospora*. Moreover, the Dothideomycetes class, which contains halophilic and halotolerant genera such as *Cladosporium* and *Aureobasidum*, was not enriched in either fraction (i.e., equal distribution in both fractions). However, two known halotolerant and halophilic species, *Hortaea werneckii* and *Aureobasidium pullulans* [45], were present in the lake sediments. In the Basidiomycota (Fig. 6B) phylum, five classes were enriched in the lysis-resistant fraction Pucciniomycetes, Microbotryomycetes, Malasseziomycetes, Exobasidiomycetes, and Cystobasidiomycetes, while the other four classes were enriched in the total fraction.

##### Diversity at the class level in the microbial mats

In the microbial mats, the Rozellomycota phylum was represented only by unidentified sequences enriched in the total fraction, similarly the Chytridiomycota phylum was represented only by unidentified sequences (enrichment in the lysis-resistant fraction). Furthermore, the classes Mortierellomycetes (Mortierellomycotina sub-phylum), Mucoromycetes (Mucoromycota phylum), Sanchytriomycetes (Monoblepharomycota phylum), and Glomeromycetes (Glomeromycotina sub-phylum) were the only identified classes in their respective phylum, and were all enriched in the total fraction. On the other hand, the Aphelidiomycetes class was enriched in the lysis-resistant fraction. In the case of the Ascomycota phylum, only the classes Taphrinomycetes and Saccharomycetes were enriched in the lysis-resistant fraction (Fig. 6C). As in the lake sediments, the Dothideomycetes class was not enriched in either fraction but, as indicated previously, the known halotolerant species *A. pullulans* [45] was present. In the Basidiomycota phylum, six of eight classes were enriched in the lysis-resistant fraction. These were, Ustilaginomycetes, Tritirachiomycetes, Microbotryomycetes, Malasseziomycetes, and Cystobasidiomycetes (Fig. 6D).

Some differences were observed when comparing the lake sediments and the microbial mats in Fig. 6 A-D. In the Ascomycota phylum, the Lecanoromycetes class was only present in the lake sediments, while the classes Taphrinomycetes and Pezizomycetes were only present in the microbial mats. Moreover, in the Basidiomycota phylum, the classes Puccinomycetes and Exobasidiomycetes were only present in the lake sediments, while the class Tritirachiomycetes was only present in the microbial mats.

#### Beyond known taxa

The predominance of unclassified sequences limited our ability to assess the effect of the lysis-resistant enrichment method on the identification of a seed bank for the fungal community. Thus, we calculated a lysis-resistant enrichment index (Fig. 6E and 6F). By calculating the enrichment of each ASV, either in the total community (index value 0–0.2) or in the lysis-resistant fraction (index 0.8–1), we were able to evaluate the effect of the lysis-resistant enrichment method even in unclassified sequences. Figure 6E and F showed that most ASVs in both environments (lake sediments and microbial mats) were either enriched in the total community or in the lysis-resistant fraction, with only a small proportion of them showing similar abundance in both fractions (index values 0.2–0.8). However, in contrast to Bacteria where OTUs would tend to be consistently found in either fraction [28], for Fungi, this is not the case and ASVs were found equally in both fractions in different environmental samples (Additional Fig. 3). Therefore, although many of these ASVs could not be classified, this analysis suggests that the lysis-resistant fungal fraction (putative seed bank) differs from the total community, but this is sample-specific.

## Discussion

### Use of the lysis-resistant separation method in Fungi

The comparison of different types of fungal structures (i.e., melanized and non-melanized asexual spores, sexual spores, melanized and non-melanized mycelia, and yeast stage) allowed us to evaluate the applicability of the lysis-resistance separation method for the fungal kingdom. Our results show that, as expected, it is more difficult to extract DNA from fungal spores than from vegetative structures (mycelia and yeast cells). Indeed, DNA was extracted from spores of *U*. *alternata*, *C*. *cinerea* and A*. niger* only after the method to enrich lysis-resistant cells. This demonstrates the utility of the enrichment method to detect fungi, especially in extreme environments where the probability to find them under the form of spores (or other resistant cells) is high. We further demonstrate that DNA extracted from mycelium is not detected using the enrichment method, but mostly using a direct DNA extraction method. This suggest that DNA detected using the enrichment method should originate from structures more resistant than typical mycelium. However, in the cases of *A*. *niger* and *U*. *alternata*, DNA from the mycelium was also not extracted using the direct DNA extraction method. This is puzzling and might indicate that the initial quantity of fungal material was probably too low. Moreover, in the case of *C*. *albicans*, our results show that non-melanized yeasts will be better detected with a direct DNA extraction method, while only a small fraction of the DNA of these yeast cells can be detected after enrichment. Therefore, the combination of both types of DNA extractions (extraction with no pre-treatment and a second extraction on the lysis-resistant fraction only) may improve the detection of fungi in different environments and broaden our understanding of the conditions that lysis-resistant fungal cells might tolerate.

### Fungal diversity in the Salar de Huasco

To the best of our knowledge, this is the first time that the diversity of the fungal community has been assessed in the Salar de Huasco. In addition, we did not restrict our analysis to the assessment of the fungal diversity overall, but we also compared this diversity to the diversity of lysis-resistant fungal cells. The latter was used as a proxy of specialized resistant cells (e.g., spores or conidia) that withstand better chemical and mechanical lysis. A large proportion of unidentified sequences was present in the data set, especially in the microbial mats. This is to be expected given that fungal diversity has been less studied than bacterial diversity. Currently, 120,000 fungal species have been described, but it is estimated that over 93% of the fungal diversity is still unknown. This suggests that the number of fungal species could be between 2.2 and 3.8 million [46]. Additionally, aquatic fungal diversity has been even less explored than fungal diversity in terrestrial environments, and studies in extreme environments such as the Salar de Huasco are rare [47]. This highlights the importance of studies such as the one presented here to better understand the distribution and diversity of fungi in the biosphere.

Our results showed that the fungal community of both the lake sediments and the microbial mats samples were dominated by members of Ascomycota and Basidiomycota, while members of basal lineages had a lower relative abundance. This is in accordance with the overall diversity currently described in fungi, with 98% of the described species belonging to the subkingdom Dikarya. Similar results have been reported regarding the fungal diversity in the Atacama Desert [48]. Furthermore, the results presented here are in agreement with a previous study assessing the fungal diversity in sediments along a salinity gradient in an estuary [49]. In the latter a high abundance of Ascomycota followed in abundance by Basidiomycota was observed at all salt concentrations. Moreover, the same study found that fresh water and medium salinity samples showed a higher abundance of basal lineages, which is also the case in our study when considering the microbial mats. The microbial mats, which in average presented a lower salinity (1.67 PSU) than the lake sediments (36.96 PSU), showed higher abundance of Chrytidiomycota, Rozellomycota, and Mortierellomycota. The only exception is Aphelidiomycota (also basal fungi, considered as a sister group to true fungi [50]), which showed higher abundance in the lake sediments than in the microbial mats. Similarly, Ascomycota and Basidiomycota were dominant in the samples with a salinity of 19.2 to 33.5 PSU in a study on the fungal diversity in the Baltic Sea [51]. These results are in agreement with the results found in the lake sediments.

### Lysis-resistance in fungi

The application of the method to enrich lysis-resistant cells to assess the diversity of fungal spores and spore-like cells, indicated an overall high similarity in the composition of the total and lysis-resistant fractions. This differs from previous studies in Bacteria, in which the production of a lysis-resistant cell appears to be restricted to some clades [52–56]. However, the assessment of the lysis-resistant community in fungi presents intrinsic challenges. For instance, all fungi produce at least one type of resistant structure within their life-cycle and their production is not only limited to survival and dispersal, but also to reproduction. Thus, the enrichment in the lysis-resistant fraction might reflect the large panel of morphological adaptations in fungal cells over the life-cycle of an individual species/taxa. Another challenge is that a large fraction of the fungal diversity is still unknown, and consequently, most of the diversity detected corresponded to unidentified sequences or sequences for which identification was only possible at high taxonomic range (for instance at the order level). Nonetheless, by calculating a lysis-resistant enrichment index of each ASV (including those unclassified), the existence of a fungal seed bank distinct from the lysis prone fraction (considered to be active vegetative cells) was evaluated. The enrichment index showed that, as in the case of Bacteria [32], most ASVs were either enriched in the lysis-resistant fraction or in the total community, with only a small proportion of them showing equal abundance in both fractions.

Although the lake sediments and microbial mats differed in biotic and abiotic factors, two phyla showed similar enrichment patterns. The Rozellomycota was enriched in the total community, while the Chytridiomycota was enriched in the lysis resistant fraction. The Rozellomycota phylum is constituted by mainly uncultured unicellular fungi, considered to be the most basal clade of fungi [57, 58]. The enrichment of this phylum in the total fraction might be due to the absence of chitin in the cell wall of most life-forms within their life-cycle, except for resting sporangia [57, 59]. The absence of a chitinous cell wall could make these cells highly susceptible to lysis. On the other hand, the Chytridiomycota phylum, enriched in the lysis-resistant fraction, is a basal phylum mainly composed of saprotrophs (degrading highly recalcitrant organic matter) and pathogens (infecting algae, plants, and amphibians) that inhabit freshwater, brackish, marine habitats and are also abundant in soils [60–62]. In the microbial mats, the sequences affiliated to Chytridiomycota could not be identified, while in the lake sediments, the Lobulomycetes class and unidentified sequences were enriched in the lysis-resistant fraction. Although little is known about the Lobulomycetes class, some members have been described as saprophytes or parasites isolated from soils [63], acidic fresh water [64], and from marine brown algae [65]. The life cycle of these fungi might provide an insight in their resistance to the lysis treatment. For most Chytridiomycota, asexual reproduction occurs through the release of zoospores, which are motile wall-less asexual spores that use a flagellum for locomotion [60]. On the other hand, when a zoospore finds a host or a substrate from which to acquire nutrients, it enters a vegetative phase in which a cell wall develops [66]. As a result, these actively metabolizing cells might be more resistant to lysis than wall-less zoospores. Additionally, some members of the Chytridiomycota are parasites of algae and rotifers, and even vascular plants [67]. In their parasitic state, they develop inside the host’s cells, and as a result, the fungal cell might be protected from lysis. A similar enrichment of intracellular bacteria was found after the application of the lysis-resistant enrichment method. The phyla Tenericutes and Chlamydiae, which are composed of intracellular pathogens [68, 69], were enriched in the lysis-resistant fraction [32].

As a whole, the most abundant phyla, Ascomycota and Basidiomycota were not enriched in either fraction in the lake sediments nor in the microbial mats. This is not surprising as, all together, these two phyla represent most of the diversity described to date. However, when considering lower taxonomic ranks, a specific enrichment of a few classes within both phyla are worth mentioning. In the Ascomycota phylum, the Saccharomycetes class was enriched in the lysis-resistant fraction of both the lake sediments and the microbial mats. This class is constituted of budding yeasts often considered to be “true yeasts”, which can undergo meiosis and sporulation under nutrient-poor conditions [70, 71]. The higher resistance of yeast to lysis and the presence of spores might explain the enrichment of Saccharomycetes in both environments. Furthermore, due to the differences of the lake sediments and the microbial mats, some of the Ascomycota classes enriched in the lysis-resistant fraction were only present in one environment. One of those is the Lecanoromycetes class, enriched in the lysis-resistant fraction of the lake sediments but absent in the microbial mats. This class is composed of lichenized fungi. The high enrichment of the Lecanoromycetes class might be due to the presence of fungal spores and or propagules (small aggregate of mycelium and phototrophic cells) used by the lichens to reproduce and disperse [72]. The two genera identified (*Caloplaca* and *Acarospora*) are not known to inhabit aquatic environments, thus the presence of lichen propagules and/or spores in the lake sediments could be linked to dispersal strategies from lichens present in the surroundings of the lake. Both these genera are worldwide distributed and have been previously found in the Atacama Desert [73, 74]. The second Ascomycota class, which was enriched in the microbial mats but was not present in the lake sediments, was Taphrinomycetes. Members of the Taphrinomycetes class are dimorphic, parasitic or pathogenic fungi [75]. The presence of fungal spores and the dimorphism of members in this class might explain their enrichment in the lysis-resistant fraction.

At the class level, more classes within Basidiomycota were enriched in the lysis-resistant fraction than for the Ascomycota phylum. The higher number of enriched classes in Basidiomycota compared to Ascomycota, might be due to their complex life cycles. Four of the six enriched classes in the Basidiomycota (Pucciniomycetes, Microbotryomycetes, Cystobasidiomycetes and Tritriachiomycetes) are members of the Pucciniomycotina subclass. This subclass accommodates a diverse array of fungi, ranging from plant pathogenic rust fungi, to insect associated fungi, mycoparasitic fungi, and dimorphic species [76, 77]. Additionally, some plant pathogens in the subclass display some of the more complex life cycles among fungi. For example, some of them are heteroecious and require two distantly related hosts to complete their life cycles, others are macrocyclic, possessing up to five distinct spore-producing states with very resistant spores such as teliospores [78].

In the lake sediments, the most enriched Basidiomycota class in the lysis-resistant fraction was the Pucciniomycetes. The Pucciniomycetes class is composed of mainly rust fungi, which are obligate plant pathogens [79]. Its enrichment in the lysis-resistant fraction in the lake sediments might reflect their presence in spore form, as no living plant was found in this hypersaline lake. Similarly, the classes Microbotryomycetes and Exobasidiomycetes, which were enriched in the lysis-resistant fraction, are also composed of plant pathogens. However, these classes might also present other ecological roles that are still unknown. In the microbial mats, the most enriched Basidiomycota class was the Tritirachiomycetes. In this class, the spores of *Paratritirachium curvibasidium* have been shown to be resistant to high temperatures (75 °C) [80]. The Ustilaginomycetes class was also enriched in the lysis-resistant fraction of the microbial mats. Members of this class are “smut fungi”, usually dimorphic, producing a saprobic haploid yeast phase and a parasitic dikaryotic hyphal phase. In their parasitic stage they occur mainly in angiosperms [81]. The enrichment of the Ustilaginomycetes class in the microbial mats might be due to the increased resistance of the bi-layered yeast walls [82] or the high resistance of spores to lysis. Furthermore some classes of the Basidiomycetes phylum were enriched in the lysis-resistant fraction of both environments, such as the lichenized class, Cystobasidiomycetes [83], and the yeast class Malasseziomycetes. Although yeast showed less resistance than spores in the validation of the enrichment method, they still showed some resistance as compared to other vegetative forms. In addition to this, the melanization of yeast in the Malasseziomycetes class [84] might increase their resistance to lysis as compared to the non-melanized yeasts of *C. albicans* used for the validation step. On the contrary, the dimorphic class Agaricostilbomycetes was enriched in the total fraction. Members of this class might have been present in this environment as mycelium and not yeast, or alternatively as non-melanized cells. These results illustrate the diversity of lysis-resistance in fungi and also that an enrichment in the total community or in the lysis-resistant fraction might better reflect the complexity of fungal life cycles rather than the presence/absence of given taxa.

## Conclusions

In conclusion, the results presented here corroborate our initial hypothesis. The widespread distribution of lysis-resistance in fungi reflects not only the presence of resistance structures, but also the multitude and complexity of life cycles in fungi. Furthermore, although lysis-resistance is broadly distributed in fungi, the application of the method to enrich for lysis-resistant cells showed that most of the ASVs are either enriched in the lysis-resistant fraction or in the total community. Thus, lysis-resistance is only characteristic for some taxa or for a particular stage in the life cycle of a given organism. In order to better understand the composition and dynamics of the fungal seedbank, molecular studies still have to be coupled with culture-dependent methods and/or microscopy to provide additional information about the morphological characteristics of the fungal lysis-resistant fraction in the environment. Regrettably, in this study, this was not possible as the amount of environmental sample was limited, which is often the case when evaluating extreme environments. Nevertheless, to the best of our knowledge this is the first study evaluating the fungal diversity in the extreme environment of the Salar de Huasco. Fungi are often overlooked in environmental studies as compared to procaryotes, especially in extreme environments. This is surprising as fungi are an integral component of every environment and representing an important resource to take into account to address major global challenges [42, 47, 85, 86]. Thus, this study does not only contribute to a better understanding of the lysis-resistance of fungal cells, but also to the unveiling of their overall diversity in extreme environments.

## Method

### Validation of the Lysis-resistant enrichment method for fungi

#### Mycelium and spore collection

The different structures for each fungal strain were produced using the growth media and temperatures summarized in Table 1. Mycelial fractions were obtained from liquid cultures agitated at 100 rpm. After effective growth, liquid cultures were transferred into 50 ml centrifuge tubes and centrifuged at 8,000 × g for 10 min. The supernatant was removed and the wet mycelial mass weighted. Then, the mycelial mass was resuspended in 5 ml 0.9% NaCl, homogenized using an ULTRA-TURRAX® stem system (IKA, Germany), before being filtered through a 0.22 μm nitrocellulose membrane (Merck Millipore, Germany) in order to retrieve only the biomass. Asexual spores were collected from fully sporulating colonies on agar-based media using 5 ml of 0.16% Tween 80 (Merck, Germany). The obtained spore suspensions were further washed through two successive cycles of: centrifugation at 5,000 × g for 5 min, supernatant removal, and pellet suspension in 5 ml 0.9% NaCl. Afterward, the number of spores collected for each fungal strain was assessed using a Neubauer chamber (depth: 0,01 mm, Carl Roth GmbH, Germany) prior to filtrating the spores through a 0.22 μm nitrocellulose membrane (Merck Millipore, Germany). Sexual spores of *C. cinerea* were collected from *in-vitro* mature fruiting bodies. A single fruiting body was immersed in 5 ml 0.9% NaCl and homogenized for a few seconds. Then, the fruiting body was removed, resulting in a suspension of sexual spores. The spore biomass of this suspension was further filtered through a 0.22 μm nitrocellulose membrane (Merck Millipore, Germany). Finally, to obtain the yeast stage of *C. albicans*, 2 colonies grown on an agar-based medium were suspended in 5 ml 0.9% NaCl before being filtered through the membrane as previously described for all other samples. All filters were kept at -20 °C before further processing. For each fungal strain, each type of biomass sample was prepared in experimental duplicates.

**Table 1:** The fungal strains, the types of samples taken as well as the growing media and conditions are listed here. Malt-extract agar (MA, malt-extract 12 g/l (SIOS Homebrewing, Switzerland), agar 15 g/l (Biolife, Italy)), Malt-extract broth (MB, malt extract 12 g/l (SIOS Homebrewing, Switzerland)), YMG (yeast extract 4 g/l (Merck, Germany), malt extract 10 g/l (SIOS Homebrewing, Switzerland), glucose 4 g/l (Merck, Germany), agar 15 g/l (Biolife, Italy)), Potato dextrose agar (PDA, 39 g/l, Carl Roth GmbH, Germany), Potato dextrose broth (PDB, potato extract 4 g/l (Merck, Germany), glucose 20 g/l (Merck, Germany)), Yeast extract potato dextrose agar (YEPDA, yeast extract 10 g/l (Merck, Germany), peptone 20 g/l (Thermo Fisher Scientific, USA), dextrose 20 g/l (Carl Roth GmbH, Germany), agar 20 g/l (Biolife, Italy), pH adjusted to 5.6). RT = room temperature

Strain	Type of sample	Growing medium	Growth temperature
Ulocladium alternata (M138)	Melanized mycelium	MB	RT
	Asexual melanized spores	MA	RT
*Aspergillus niger* (M8)	Mycelium	MB	30 °C
	Asexual	MA	30 °C
*Coprinopsis cinerea* (M141)	Mycelium	MB	RT
	Sexual spores (fruiting body)	YMG	30 °C
	Asexual spores	YMG	30 °C, in the dark
*Mortierella antarctica* (M162)	Mycelium	PDB	RT
	Asexual spores	PDA	RT
*Candidas albicans*	Yeast form	YEPDA	37 °C

#### Method to enrich lysis-resistant cells

To one-half filter containing biomass, 900 µl of 1 × TE (Tris–EDTA) buffer were added and the biomass was resuspended by vortexing. The second half of the filter was used for DNA extraction without enrichment. For the enrichment, the sample was incubated at 65 °C for 10 min under constant agitation at 80 rpm. Afterwards, 100 µl of 20 mg/ml lysozyme were added and the sample was incubated at 37 °C for 60 min under constant agitation at 80 rpm. Later, 250 µl of 3 N sodium hydroxide (NaOH) and 250 µl of 6% sodium dodecyl sulfate (SDS) solutions were added and the sample was incubated at room temperature for 60 min under constant agitation at 80 rpm. Next, the sample was filtered on a 0.22 µm sterile nitrocellulose filter, which was then washed twice with 2 ml of sterile physiological water to remove any residue of the solutions used. The filter was then left to dry under a laminar flow hood. Once the filter was dry, 450 µl of sterile ultrapure water, 50 µl of 1 × DNase reaction buffer, and 0.5 µl Dnase enzyme were added and incubated at room temperature for 15 min directly on the filter, to degrade the DNA released by the easy-to-lyse cells prior to the DNA extraction from the lysis-resistant fraction. After 15 min, the solution was filtered and the residue was washed once more with 1 ml of physiological water to wash away any degraded DNA as well as the DNAase solution. Finally, the filters (containing the lysis resistant fraction only) were stored at -20 °C until the DNA extraction [27].

#### DNA extraction

First of all, the same DNA extraction method was used for both fractions, the total community (i.e., no pretreatment to remove lysis-resistant cells) and the lysis-resistant fraction (Additional Fig. 4). The FastDNA®SPIN kit for soil (MP Biomedicals, USA) was used with a modified protocol that included three successive bead-beating rounds in the first step in order to maximize the amount of DNA recovered. All three DNA fractions obtained from the same sample were pooled together at the end of the DNA extraction as described previously [27], then precipitated with ethanol, and resuspended in PCR-grade water. Finally, the DNA was quantified using the Qubit® dsDNA HS Assay Kit on a Qubit® 2.0 Fluorometer (Invitrogen, Carlsbad, CA, USA).

Additionally, microscopy observations were done after the enrichment for the filters in which the treatment was applied or before DNA extraction for the other filters in order to assess the effect of the enrichment method on the mycelial, the spore/yeast fractions. For this, the biomass contained on one half of a filter was resuspended in 1 ml of TE-Buffer. A drop of the suspension obtained was then observed using an Upright Leica DM4 B Microscope (Leica, Wetzlar, Germany) and images were generated with a Leica Microscope Camera DFC7000T (Leica, Wetzlar, Germany).

### Application of the lysis-resistant enrichment method in environmental samples

#### Sampling

The sampling at Salar de Huasco took place in September of 2019 and all samples were collected within 24 h. The lake sediment samples were collected following a saline gradient, with salinity ranging from 53 to 0.706 PSU (Fig. 2A). Seven sediment core samples were collected from the main saline lake, with a core-borer of approx. 4 cm in diameter and approx. 7 cm in depth. A sterile core-borer was pushed manually into the sediment and sealed after collection of the sample. Sediment was extracted from the corer under sterile conditions. The cores were then cut on site to 1 cm-thick subsamples and stored in sterile plastic Petri dishes. In addition, 40 ml of microbial mats were collected in sterile 50 ml Falcon tubes from eighteen small ponds surrounding the main saline lake. At every sampling point, salinity, pH, conductivity, water temperature, and dissolved oxygen were measured using a Multiparameter Water Quality Meter-HI98194 (Hanna Instruments, Rhode Island, USA) and the coordinates and altitude were recorded (Additional Table 2). The samples were transported back to the laboratory at room temperature (approx. 20–25 °C), where they were stored at 5 °C until being processed a week later.

#### Lysis-resistant cells enrichment method

Before applying the method to enrich lysis-resistant cells to environmental samples one additional step was added to the procedure described in Method to enrich lysis-resistant cells section To avoid any bias during the enrichment of the lysis-resistant fraction due to the attachment of cells to mineral particles, an indirect DNA extraction method was used [27]. First, 3 g of material from each sample were weighted in ULTRA-TURRAX® disperser tubes. Then, 15 ml of Na-Hexametaphosphate (1%) were added and the mixture was homogenized two times at 3′000 rpm for 1 min with the ULTRA-TURRAX® tube disperser workstation system (IKA, Germany). The sample was left to sediment for 10 min and the supernatant was collected. This initial step was repeated twice. The two 15 ml supernatants were pooled, centrifuged at 20 × g for 1 min to precipitate large particles, and the remaining supernatant was filtered through a 0.2 µm sterile nitrocellulose filter. For each sample, the filter was cut in half. One half was used to assess the total fungal community by directly extracting DNA and the second half was subjected to the enrichment method, thereby assessing only the lysis-resistant fraction. This way, the same filter was used to assess both type of communities (Additional Fig. 3). The enrichment method and DNA extraction were performed as already described in Method to enrich lysis-resistant cells section. The only difference was that only half a filter containing the biomass was used for the enrichment method in environmental samples, as compared to a full filter for the validation samples.

### Sequencing and statistical analysis

The purified DNA extracts were sent to Fasteris (Geneva, Switzerland) for targeted amplicon sequencing of the fungal ITS2 region using an Illumina MiSeq platform (Illumina, San Diego, CA, USA), generating 300 bp paired-end reads. The ITS2 region was amplified using the universal primers ITS3 KYO2 (5’-GAT GAA GAA CGY AGY RAA-3’) and ITS4 (5’-TCC TCCGCT TAT TGA TAT GC-3’) [87]. Demultiplexed and trimmed sequence reads provided by Fasteris were processed using QIIME2 [88] with dada2 [89] for the denoising step. Read lengths were truncated to optimized total nucleotide lengths (based on q-scores) at 478 bases total composite length for the un-joined sequences. These truncated sequences allowed the joining of denoised paired-end reads by at least 12 identical bases to obtain full denoised sequences length of 466 bases. Sequences were grouped on Amplicon Sequence Variants (ASVs). The ASVs obtained were then taxonomically classified using QIIME2’s vSEARCH-based consensus taxonomy classifier [90] with the UNITE database version 8.2, release date February 4, 2020 [91].

All statistical analysis were performed with RStudio version 1.3.1093 [92], the community and multivariate analysis were performed with the phyloseq [93] and vegan [94] packages. The Venn Diagram was calculated with the package Venn.Diagram [95]. The relative abundance was calculated using the Total-Sum Scaling (TSS) normalization. The principal coordinate analysis (PCoA) was calculated based on the Bray–Curtis distances. For the Venn Diagram, PCoA and the enrichment analysis, the relative abundance was used. For the statistical test of the diversity using the Shannon–Weaver and the Simpson diversity indices and the analysis of variance (ANOVA) the raw abundance data was used. The proportion used in the distribution plots was calculated by phylum, the relative abundance of each ASV was divided by the sum of all relative abundance to obtain a proportion, the log base 10 of the proportion was used for the graphical representation. The enrichment proportion was calculated by first adding the relative abundance per genus once in the total fraction and once in the lysis-resistant fraction. The proportion was then calculated by subtracting the lysis-resistant abundance from the total abundance and dividing this by the sum of the lysis-resistant fraction and the total fraction.

Additional analyses were performed to evaluate the contribution of unclassified ASVs to the composition of the lysis-resistant community. For this, an “enrichment index” was calculated for each individual ASV by comparing the relative contribution of the abundance in the lysis-resistant fraction (based on absolute total sequence counts) as compared to the total abundance of the ASV (lysis/(lysis + total fraction)). At the end, an index ranging from 0 (ASVs only detected in the total community fraction) to 1 (ASVs only detected in the lysis-resistant fraction) is obtained for each environmental sample. The results were represented as cumulative graphs, in which the proportion of ASVs displaying the index value were grouped together.

## Supplementary Information

**Additional file 1:** **Figure 1.** Lake sediments 50 most abundant ASVs per sampling point at the phylum level. Left, lysis-resistant fraction and right total community, different colors represent different phyla.

**Additional file 2:** **Figure 2.** Microbial mats 50 most abundant ASVs per sampling point at the phylum level. Left, lysis-resistant fraction and right total community, different colors represent different phyla.

**Additional file 3:** **Figure 3.** Heatmap representing the enrichment index calculated for the ASVs for which the enrichment index can be calculated in all individual samples (most prevalent ASVs)

**Additional file 4:** **Figure 4.** Graphical representation of the method used. Left, graphical description of the validation method and right, graphical representation of the method used for the analysis of the fungal community.

**Additional file 5:** **Table 1.** Raw values of the DNA quantification after DNA extraction. DNA concentration in ng/µl.

**Additional file 6:** **Table 2.** Information on the abiotic parameters measured in situ for the different sampling locations. %OD= dissolved oxygen.

## Data Availability

The dataset generated and analyzed during the current study is available in the GenBank (NCBI) repository under the following submission number: PRJNA853532. Accession link: https://www.ncbi.nlm.nih.gov/bioproject/PRJNA853532.
