# Supplementary figures and images for: Assessment of fungal spores and spore-like diversity in environmental samples by targeted lysis
Source: BMC Microbiol. 2023 Mar 14;23:68. doi: 10.1186/s12866-023-02809-w (PMC10015814; doi:10.1186/s12866-023-02809-w)

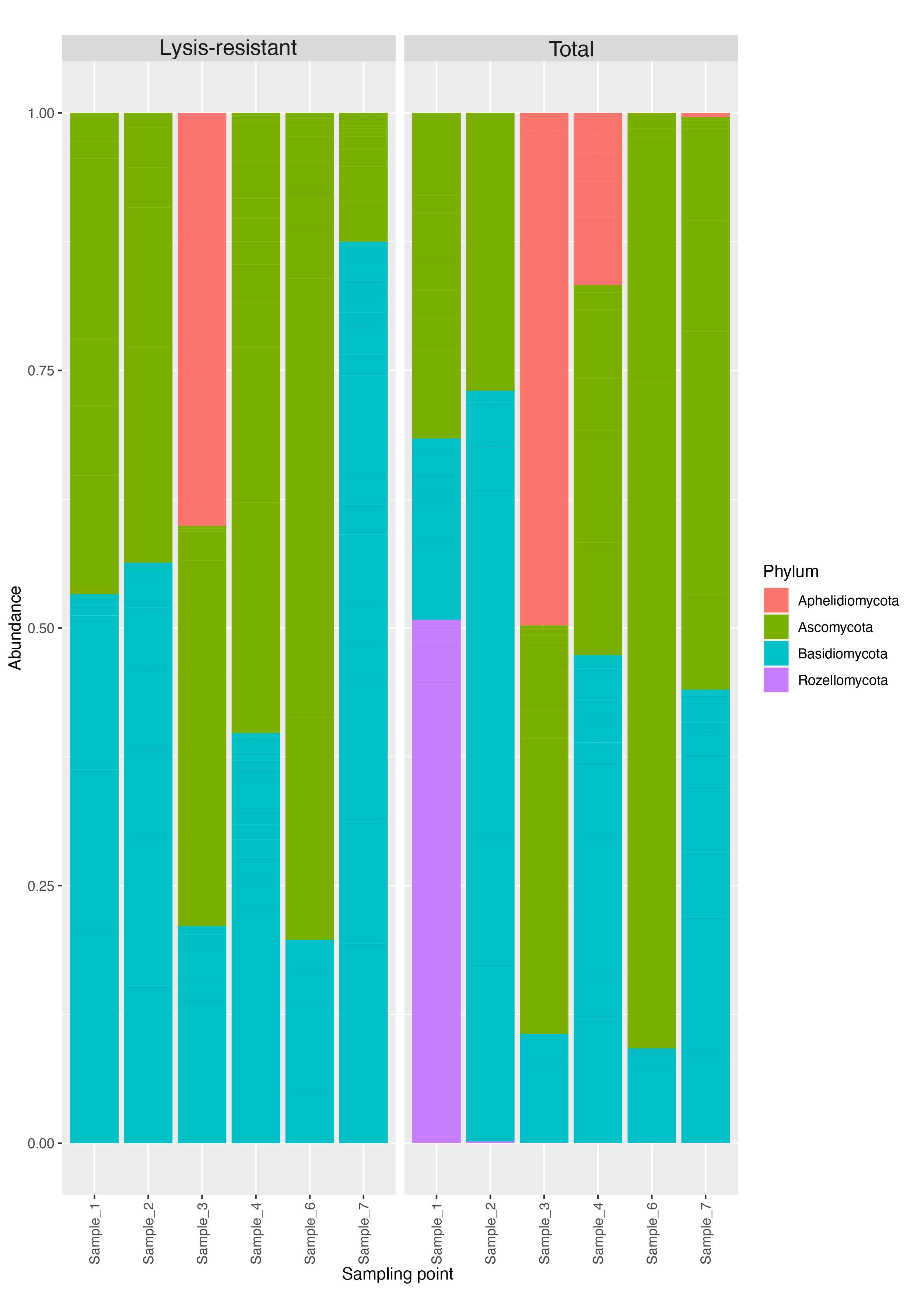

Supplement: Supplementary file 1 — Additional file 1: Figure 1. Lake sediments 50 most abundant ASVs per sampling point at the phylum level. Left, lysis-resistant fraction and right total community, different colors represent different phyla. [file 12866_2023_2809_MOESM1_ESM.png]

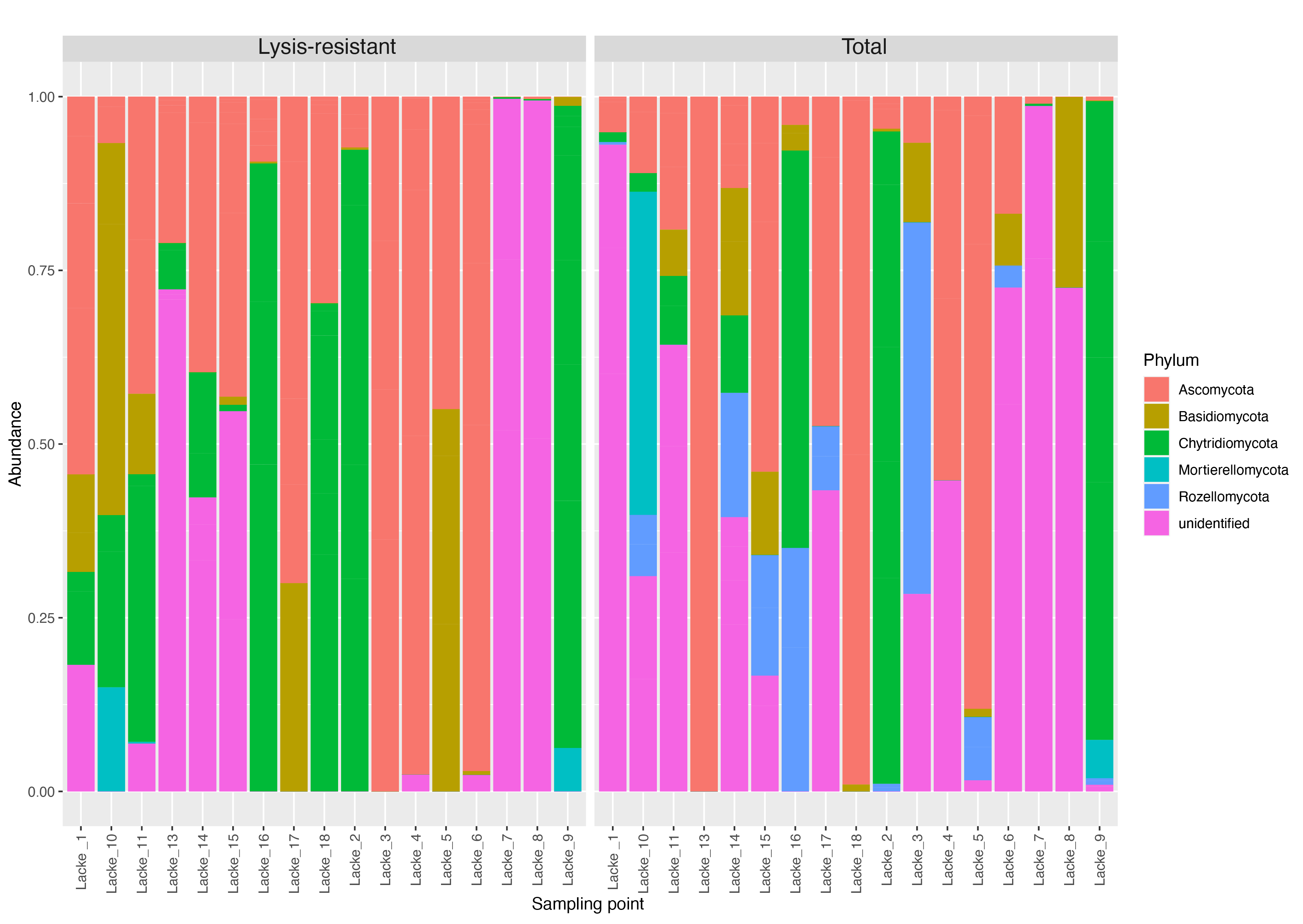

Supplement: Supplementary file 2 — Additional file 2: Figure 2. Microbial mats 50 most abundant ASVs per sampling point at the phylum level. Left, lysis-resistant fraction and right total community, different colors represent different phyla. [file 12866_2023_2809_MOESM2_ESM.png]

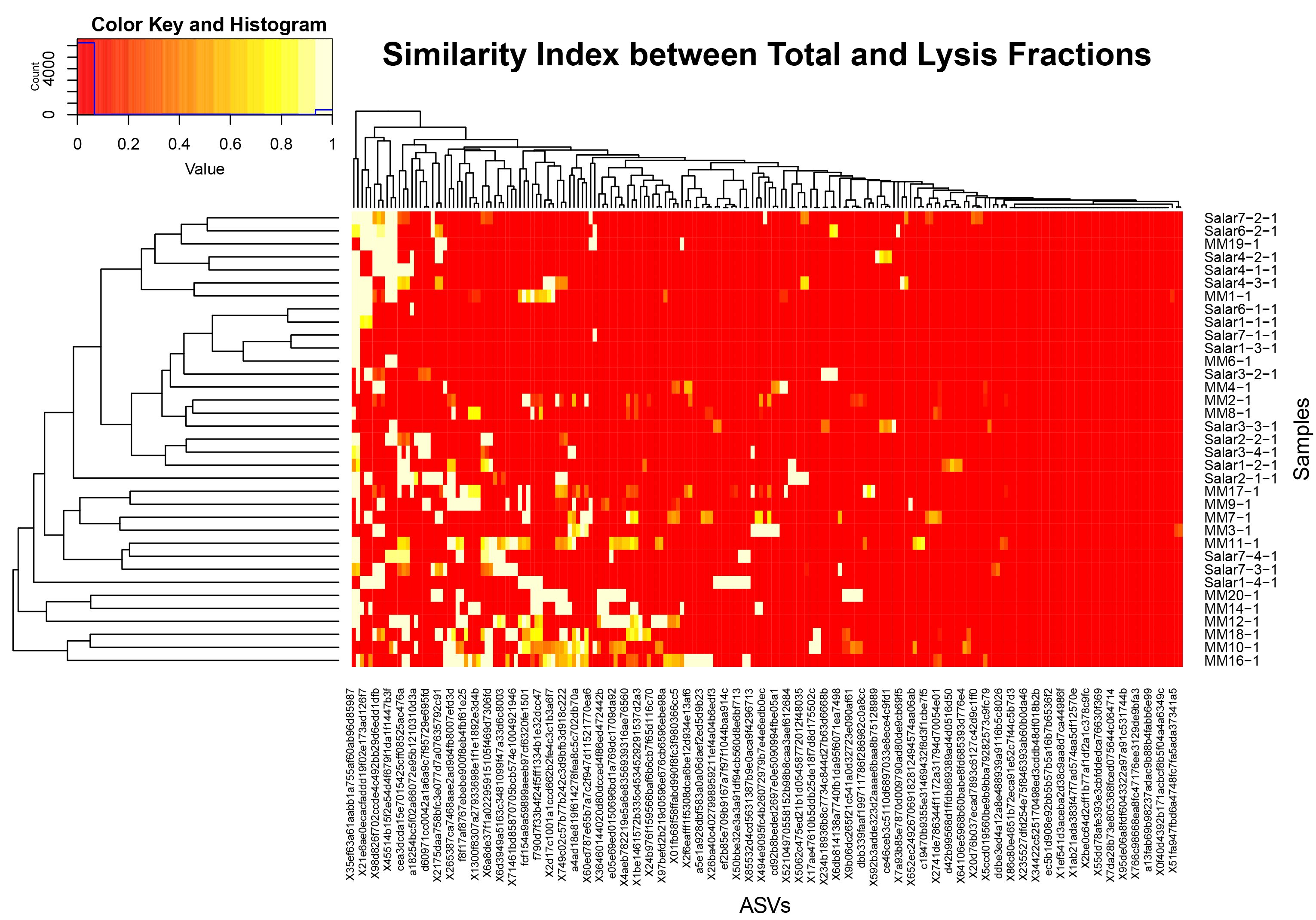

Supplement: Supplementary file 3 — Additional file 3: Figure 3. Heatmap representing the enrichment index calculated for the ASVs for which the enrichment index can be calculated in all individual samples (most prevalent ASVs) [file 12866_2023_2809_MOESM3_ESM.png]

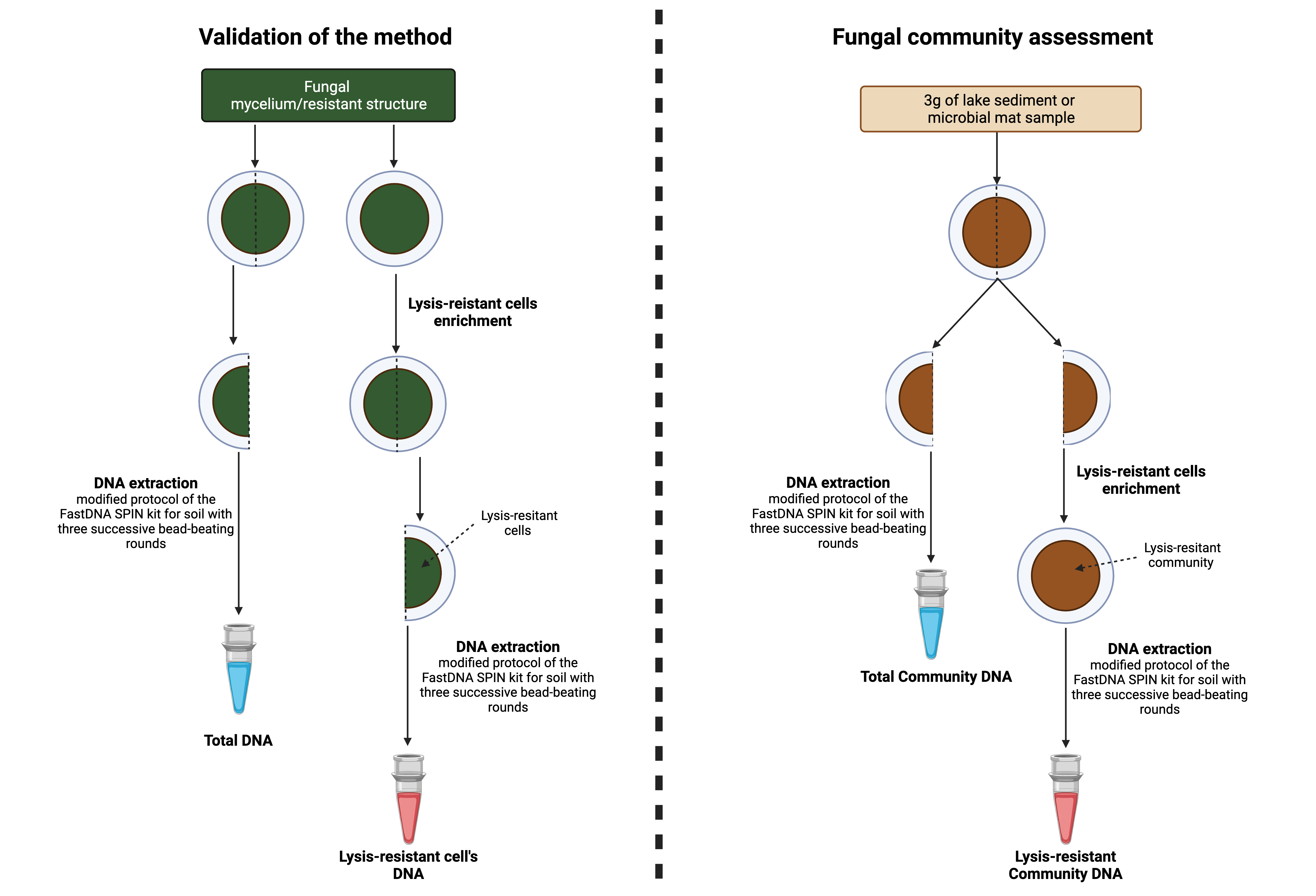

Supplement: Supplementary file 4 — Additional file 4: Figure 4. Graphical representation of the method used. Left, graphical description of the validation method and right, graphical representation of the method used for the analysis of the fungal community. [file 12866_2023_2809_MOESM4_ESM.png]
